# Polypharmacy in Type 2 Diabetes Mellitus: Insights from an Internal Medicine Department

**DOI:** 10.3390/medicina55080436

**Published:** 2019-08-03

**Authors:** Elena-Codruța Dobrică, Mihnea-Alexandru Găman, Matei-Alexandru Cozma, Ovidiu Gabriel Bratu, Anca Pantea Stoian, Camelia Cristina Diaconu

**Affiliations:** 1 “Carol Davila” University of Medicine and Pharmacy, 8 Eroii Sanitari Boulevard, Bucharest 050474, Romania; 2 Emergency University Central Military Hospital, 88 Mircea Vulcanescu Street, Bucharest 010825, Romania; 3 Academy of Romanian Scientists, 54 Splaiul Independentei Street, Bucharest 030167, Romania; 4 Internal Medicine Clinic, Clinical Emergency Hospital of Bucharest, 8 Calea Floreasca Street, Bucharest 014461, Romania

**Keywords:** diabetes mellitus, polypharmacy, drug-drug interactions, food-drug interactions, comorbidities, multimorbidity, elderly

## Abstract

*Background and Objectives*: Polypharmacy heavily impacts the quality of life of patients worldwide. It is a necessary evil in many disorders, and especially in type 2 diabetes mellitus, as patients require treatment both for this condition and its related or unrelated comorbidities. Thus, we aimed to evaluate the use of polypharmacy in type 2 diabetes mellitus vs. non-diabetes patients. *Materials and Methods*: A cross-sectional retrospective observational study was conducted. We collected the medical records of patients hospitalized in the Internal Medicine Clinic of the Clinical Emergency Hospital of Bucharest, Romania, for a period of two months (01/01/2018–28/02/2018). Patients diagnosed with type 2 diabetes mellitus were included in the study group, whereas patients who were not diabetic were used as controls. *Results*: The study group consisted of 63 patients with type 2 diabetes mellitus (mean age 69.19 ± 9.67 years, range 46–89 years; 52.38% males). The control group included 63 non-diabetes patients (mean age 67.05 ± 14.40 years, range 42–93 years, 39.68% males). Diabetic patients had more comorbidities (10.35 ± 3.09 vs. 7.48 ± 3.59, *p* = 0.0001) and received more drugs (7.81 ± 2.23 vs. 5.33 ± 2.63, *p* = 0.0001) vs. non-diabetic counterparts. The mean number of drug-drug and food-drug interactions was higher in type 2 diabetes mellitus patients vs. controls: 8.86 ± 5.76 vs. 4.98 ± 5.04, *p* = 0.0003 (minor: 1.22 ± 1.42 vs. 1.27 ± 1.89; moderate: 7.08 ± 4.08 vs. 3.54 ± 3.77; major: 0.56 ± 0.74 vs. 0.37 ± 0.77) and 2.63 ± 1.08 vs. 2.19 ± 1.42 (*p* = 0.0457), respectively. *Conclusions*: Polypharmacy should be an area of serious concern also in type 2 diabetes mellitus, especially in the elderly. In our study, type 2 diabetes mellitus patients received more drugs than their non-diabetes counterparts and were exposed to more drug-drug and food-drug interactions.

## 1. Introduction

Although it is an important health issue in clinical practice, a universal definition of polypharmacy has yet to be established. Most commonly, it is agreed that “the use of 5 or more medicines every day” is equivalent to polypharmacy [1]. However, polypharmacy is not only characterized by an increased daily intake of drugs, but also by adverse effects, drug-drug and drug-food interactions, as well as prescription of unnecessary medication [2]. It is a phenomenon with an ascending prevalence due to a rise in the average lifespan and also due to the presence of a high number of comorbidities in many patients. In many instances, an additional comorbidity requires at least one additional therapeutic agent. Also, polypharmacy often leads to the development of a vicious circle: An increased number of medicines causes adverse events that are mistakenly interpreted as additional disorders or comorbidities and lead to the prescription of additional drugs [3]. Moreover, physicians must be able to discriminate between “appropriate” and “inappropriate” polypharmacy. The distinction between the two is based on the necessity to prescribe more drugs to a single patient and on the risk–benefit ratio [4].

Type 2 diabetes mellitus (T2DM) is a considerable cause of polypharmacy, explained by the necessity to treat microvascular and macrovascular complications, but also due to the presence of comorbidity clusters. Elderly diabetic patients are at particular risk for polypharmacy for various reasons: Multimorbidity (arterial hypertension, solid or hematological malignancies, chronic heart failure, etc.), age-related pharmacokinetic variability in the setting of liver or kidney diseases, lack of adherence (voluntary or involuntary—in psychiatric disorders), and others. Also, elderly patients are more likely to take over-the-counter medications and herbal supplements, which can lead to drug interactions [5,6,7,8,9,10]. Moreover, suboptimal management of diabetes is frequent in the elderly who are, in many instances, homebound and are unable to control their disease in a proper manner. This increases their risk of both hypo- and hyperglycemia, which are also factors that lead to polypharmacy in advanced age [11]. Poor self-care directly influences elderly patients’ ability to achieve glycemic control, since it renders the patients unable to appropriately self-manage their disease [12].

The objective of our study was to investigate the employment of polypharmacy and the number of drug-drug and drug-food interactions in T2DM patients hospitalized in the Internal Medicine Clinic of the Clinical Emergency Hospital of Bucharest, Romania. To our knowledge, this issue of utter importance has not been explored in our country so far.

## 2. Materials and Methods

### 2.1. Study Design

We conducted a cross-sectional observational retrospective study to evaluate the employment of polypharmacy and the number of drug-drug and drug-food interactions in T2DM patients. In order to assess the employment of polypharmacy and the number of drug-drug and food-drug interactions in diabetic patients, we retrospectively analyzed the medical records of all the patients aged ≥18 years who were treated in the Internal Medicine Clinic of the Clinical Emergency Hospital of Bucharest, a tertiary reference center in Bucharest, Romania, between 1 January 2018 and February 28, 2018 (two-month period). The Clinical Emergency Hospital of Bucharest is one of the largest teaching hospitals in Romania, providing care services for a variety of medical and surgical specialties, predominantly to residents in Bucharest, the capital of Romania, but also to patients from the entire country referred to this facility.

As a first step in our research, we collected data regarding the age, sex, comorbidities, and medications prescribed to these patients, and a Microsoft Office Excel database was created using these variables. Patients who had incomplete medical records were excluded. The second step of our research consisted of splitting the patients into two groups: A group of patients who suffered from T2DM (study group) and another group of patients who attended our Internal Medicine Clinic for other illnesses (control group). Afterwards, we calculated the number of comorbidities for each patient, analyzed the types of drugs prescribed and identified the drug class for each medication, and calculated the number of drugs for each patient as well as the type and number of drug-drug and food-drug interactions to assess the employment of polypharmacy. Drug-drug and food-drug interactions were checked using an online program (the Drug Interactions Checker, available at https://www.drugs.com/drug_interactions.html). The Drug Interactions Checker requires the user to type the name of the drug and select it from a list in order to confirm the choice. Multiple drugs can be searched for at the same time and, when the list is complete, by pressing the Check for Interactions button, a Drug Interaction Report is shown on the computer screen. The Drug Interaction Report classifies the results into major, moderate, and minor drug-drug interactions, as well as food (for food-drug interactions) and therapeutic duplications. For example, the concomitant prescription of warfarin and omeprazole leads to a moderate drug-drug interaction between warfarin and omeprazole, and a food-drug interaction between warfarin and certain foods rich in vitamin K. The prescription of aspirin and omeprazole leads to a minor drug-drug interaction, whereas the prescription of aspirin and ibuprofen leads to a major drug-drug interaction. After obtaining these results, the database was completed with the number and type of drug interactions for each patient. We defined polypharmacy as “the use of 5 or more medicines every day” in a single patient [1].

### 2.2. Setting

The study was conducted using medical records of the Internal Medicine Clinic of the Clinical Emergency Hospital of Bucharest, Romania.

### 2.3. Participants

A total of 63 T2DM patients were included in the study, representing all patients diagnosed with T2DM who were referred to the Internal Medicine Clinic between 1 January and 28 February 2018. The records of the 63 T2DM patients were compared to the records of 63 randomly selected non-diabetic patients referred to our department for other afflictions, who consisted the control group of the study.

### 2.4. Data Sources and Variables

Medical records were employed to retrieve data and to create a new database with the following variables: Sex, age, comorbidities, prescribed drugs, and drug-drug or food-drug interactions. The study group consisted of all the T2DM patients who were referred to the Internal Medicine Department during the study period. A control group was randomly selected from the patients who were admitted to the Internal Medicine Department but did not suffer from T2DM. 

### 2.5. Bias

To reduce selection bias, we included all patients aged ≥18 years with complete medical records attending our department during the study period. Patients with incomplete medical records or deceased during hospitalization were excluded.

### 2.6. Statistical Methods

Categorical variables were presented as frequencies and percentages. Continuous variables were presented as the mean ± SD. Patients were divided according to the presence and absence of T2DM. Continuous variables were compared using independent samples t-test. The level of significance was presented as *p*-values in different tables. The analysis was performed at a 5% level of significance using Microsoft Excel (Microsoft Office Professional Plus 2013), MedCalc (https://www.medcalc.org) and GraphPad QuickCalcs (https://www.graphpad.com). Post-hoc power analyses were run to verify the power of these retrospective findings using https://clincalc.com/stats/Power.aspx.

### 2.7. Ethical Standards

All patients signed at admission an informed consent, agreeing to partake in research projects as long as their confidentiality was respected. All procedures of this study respect the ethical standards of the Helsinki Declaration of 1975, as revised in 2008(5), as well as the national law and the guidelines of the hospital ethics council who approved the research (approval number 4263/13.05.2019).

## 3. Results

A total of 126 patients were enrolled: 63 T2DM patients (mean age 69.19 ± 9.67 years, range 46–89 years, 52.38% males) and 63 non-T2DM patients (67.05 ± 14.40 years, range 42–93, 39.68% males). T2DM patients were older vs. non-diabetic controls (*p* = 0.0189). The number of comorbidities was assessed for each group and the results were as following: T2DM patients had more comorbidities (10.35 ± 3.09 vs. 7.48 ± 3.59, *p* = 0.0001; post-hoc power = 99.8%) and received more drugs vs. non-T2DM counterparts (7.81 ± 2.23 vs. 5.33 ± 2.63, *p* = 0.0001; post-hoc power = 100.0%). The mean number of drug-drug interactions was higher in T2DM patients vs. controls (8.86 ± 5.76 vs. 4.98 ± 5.04, *p* = 0.0003; post-hoc power = 98.0%). Food-drug interactions were also higher in T2DM patients vs. controls (2.63 ± 1.08 vs. 2.19 ± 1.42, *p* = 0.0457; post-hoc power = 49.9%). In terms of severity, moderate drug-drug interactions were more frequent in T2DM patients vs. controls (7.08 ± 4.08 vs. 3.54 ± 3.77, *p* = 0.0001; post-hoc power = 100.0%). The differences between groups were not statistically significant in terms of minor drug-drug interactions (1.22 ± 1.42 vs. 1.27 ± 1.89, *p* = 0.8774) or major drug-drug interactions (0.56 ± 0.74 vs. 0.37 ± 0.77, *p* = 0.1873) (Table 1).

**Figure 1:**
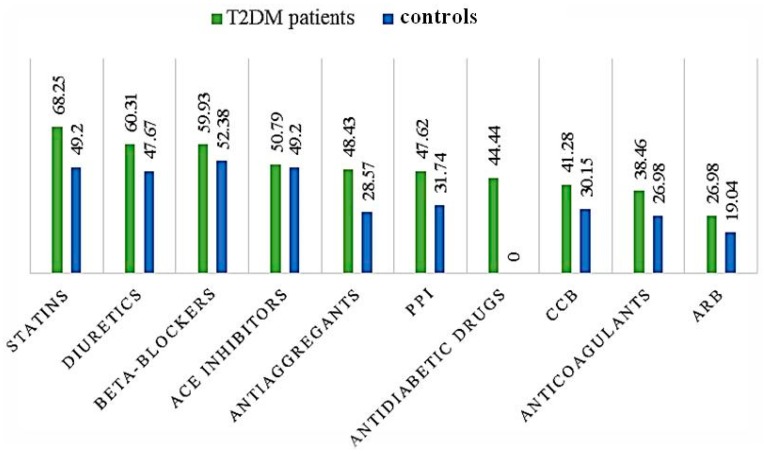
Most common drug classes (expressed in %) prescribed in patients with type 2 diabetes mellitus (T2DM) vs. controls (ACE—angiotensin-converting enzyme; PPI—proton-pump inhibitors; CCB—calcium channel blockers; ARB—angiotensin II receptor antagonists).

In the T2DM group, the most common drug classes prescribed were statins, diuretics, beta-blockers, and angiotensin-converting enzyme (ACE) inhibitors, with more than half of the study group having been prescribed such drugs. In controls, beta-blockers were the most prescribed, followed by statins, ACE inhibitors, and diuretics (Figure 1).

Anti-diabetic medication was prescribed in 53.13% (*n* = 34) of cases, with metformin and gliclazide as the most frequent agents employed alone or in combination with other drugs. A percentage of 38.25% (*n* = 13) of the diabetic patients who received medication required combination therapy of at least two anti-diabetic agents to control their disease. The prescription patterns of anti-diabetic medication reported are: Metformin alone = 38.25% (*n* = 13), insulin alone = 14.71% (*n* = 5), metformin + gliclazide = 11.76% (*n* = 4), metformin + insulin 8.82% (*n* = 3), metformin + glimepiride 5.88% (*n* = 2), gliclazide alone 5.88% (*n* = 2), gliquidone 2.94% (*n* = 1), metformin + glibenclamide 2.94% (*n* = 1), metformin + pioglitazone 2.94% (*n* = 1), gliclazide + acarbose 2.94% (*n* = 1), and metformin + gliclazide + sitagliptin 2.94% (*n* = 1) (Table 2).

Drug-drug and food-drug interactions as reported by the online program can be:

- Major = the interaction possesses a significant clinical value and should be avoided since there are more risks *versus* benefits.
- Moderate = the interaction possesses a moderate clinical significance and should usually be avoided or used only if necessary.
- Minor = the interaction possesses a minimal clinical significance, but in order to minimize any risks for the patient, an alternative drug should be considered, or the patient should be carefully monitored.

The most common drug-drug and food-drug interactions encountered in the T2DM study group and in the control group are reported in Table 3 and Table 4.

## 4. Discussion

The objective of our study was to evaluate the impact of T2DM on the number of drugs prescribed to diabetic patients aged ≥18 years and the impact of polypharmacy on diabetic patients with respect to the number of drug-drug and drug-food interactions. We compared patient characteristics and polypharmacy in 63 T2DM patients admitted to the Internal Medicine Department of a referral emergency hospital in Bucharest, Romania, and in 63 non-diabetic subjects randomly selected from the patients who were admitted to the same department for diseases other than diabetes. Our results suggest that diabetic subjects have more comorbidities and are prescribed more drugs in comparison to their non-diabetic counterparts. Our data clearly shows that, irrespective of drug class, a higher percentage of T2DM patients received medicines than patients referred to the hospital for other conditions. Also, there are differences in the pattern of medications most commonly prescribed: In the first group, the most frequent drugs prescribed were statins (68.25%), followed by diuretics (60.31%). On the other hand, in the control group, the most frequent medicines prescribed were beta-blockers (52.38%), followed by ACE inhibitors and diuretics (49.20% each).

We also noted a difference in the mean number of drug-drug interactions: Diabetic subjects were approximately two times more likely to be exposed to drug-drug interactions in comparison with non-diabetic patients. Elderly patients with diabetes are more likely to experience adverse reactions to drugs as opposed to younger patients with diabetes. In the elderly, the metabolic pathways that are involved in drug breakdown are altered and drug clearance is significantly reduced if kidney and/or liver disorders are present. Also, Noale et al. reported that drug interactions are more likely encountered in certain groups of patients: Females, patients who inadequately control their caloric intake, and patients who show a poor management of their diabetes [8]. The main single agents prescribed in the management of diabetes were metformin and, in a lower percentage, gliclazide. Combination therapy of at least two anti-diabetic drugs was employed to control glucose levels in almost 39% of patients. Combination therapy has been widely used in the last decades in the therapy of diabetes, increasing the risk of polypharmacy and adverse drug reactions nevertheless, since many physicians prefer to prescribe an additional drug rather than change the class of anti-diabetic agents [13,14]. In a study conducted on 220 diabetic patients, Singh et al. reported adverse drug reactions in 12.86% patients on biguanides, 19.05% in patients on sulphonylureas, and 11.76% in patients treated with combination of biguanide + sulphonylurea [14].

Our results reinforce that polypharmacy is a serious concern in T2DM patients. To our knowledge, this is the first Romanian study aiming to explore the employment of polypharmacy in diabetic subjects. Similar recently published studies have suggested that polypharmacy is often encountered in the management of diabetes due to multimorbidity and the necessity to treat a wide spectrum of co-occurring disorders. A cross-sectional retrospective observational study of 8932 type 2 diabetic patients, performed by Alwhaibi et al. in an outpatient clinic, found out that almost 78% of the patients were exposed to polypharmacy, with women being more affected than men. The risk of polypharmacy was two times higher in patients with cardiovascular comorbidities, respiratory diseases, and mental disorders, and three times higher in those with musculoskeletal diseases. Also, patients with osteoarthritis frequently require the use of analgesics and NSAIDs for chronic pain, drugs which are associated with side effects such as increased blood pressure values, gastrointestinal hemorrhages, increased risk of myocardial infarction, and others [15]. The vast majority of patients with T2DM are elderly patients with multiple comorbidities, many of which are complications of their neglected and inappropriately managed diabetes. In a research project conducted in several Swiss university primary care centers on 1002 patients of whom 292 (29.1%) suffered from diabetes, Aubert et al. discovered a strong association between polypharmacy and diabetes mellitus (odds ratio = 4.47; 95% confidence interval = 3.23 − 6.20). The only comorbidity for which the association with polypharmacy was stronger was hypertension (odds ratio = 8.49; 95% confidence interval = 5.25 − 13.73) [16].

One Canadian study, which enrolled nursing home residents, reported polypharmacy employment in 214 patients with type 2 diabetes mellitus and arterial hypertension, with 48% of these patients having been prescribed at least nine drugs. The polymedicated patients were more likely to have an established diagnosis of arterial hypertension or congestive heart failure, but less likely to have dementia. More non-antidiabetic drugs were prescribed in patients with overtreated diabetes (those receiving at least one antidiabetic drug, with a HbA1c ≤ 7.5%). The authors concluded that the aggressive treatment of cardiovascular risk factors raises the risk of polypharmacy, especially in frail patients [17]. A rational medication prescription is necessary in diabetic patients in order to achieve the therapeutic goals with the fewest drugs possible. The strategies to fight against polypharmacy focus on “inappropriate” drugs. However, the use of “appropriate” drugs, recommended by the international guidelines, can result in severe side effects that often require presentation to the emergency room and/or hospitalization. Shehab et al. reported that 4 in 1000 visits to the emergency department in the United States are due to adverse drug events, out of which 27.3% require hospitalization. The patients most affected by adverse drug events were aged ≥65 years, and this age group accounted for 34.5% of the visits to the emergency room and for 43.6% of the hospitalizations. Of the emergency department presentations for adverse drug events, approximately 46.9% were due to intake of blood thinners (causing hemorrhage), antibiotics (causing moderate or severe allergic reactions), and anti-diabetic medication (causing hypoglycemia and moderate-severe neurological disturbances). In the group older than 65 years, these drugs accounted for 59.9% of the arrivals to the emergency room [18]. Another study reported that 15.2% of the arrivals to the emergency department in patients over 80 years old are due to anti-diabetic agents [19]. This highlights the importance of a careful therapeutical management of common drugs in the elderly, not only of focusing on “inappropriate” drugs. Since arterial hypertension, dyslipidemia, chronic heart failure, atrial fibrillation, obesity, chronic kidney disease, and neoplastic disease are common comorbidities in T2DM patients that request medical therapy and appropriate management, physicians should make appeal to therapeutic solutions with a lower risk of drug-drug and food-drug interactions when available (e.g., non-vitamin K antagonists oral anticoagulants could be selected over vitamin K antagonists to prevent thromboembolic events in patients with atrial fibrillation, more research should be conducted to explore the potential uses of phytochemicals with clinical benefits, fewer drug-drug interactions and antioxidant properties, reconsideration of primary prevention strategies, etc.) [20,21,22,23,24,25,26]. In a similar study, Geitona et al. reported that diabetic patients younger than 65 years were less likely to exhibit polypharmacy as compared to diabetic patients aged 66 or more (*p* = 0.005). Of the latter, 60.1% were prescribed five or more drugs daily. Likewise, comorbidities played a decisive role in the decision to prescribe polypharmacy in diabetic patients. In their study, diabetic patients with multimorbidity suffered from polypharmacy in 44.4% of cases as compared to only 4.8% of patients who had diabetes alone (*p* = 0.001). Also, diabetic patients were more inclined to take medication rather than follow dietary or physical exercise recommendations provided by their attending physician [27].

Polypharmacy is common also in patients with diabetes living in nursing homes. A study involving 75 diabetic nursing home residents from Coventry, United Kingdom, revealed that 84% (*n* = 63) were affected by polypharmacy. The most common drug classes prescribed to these patients were anti-platelet drugs (59%, *n* = 44), such as aspirin, clopidogrel or dipyridamole, and statins (41%, *n* = 31). In 24% (*n* = 18) of the study group, the price of the medications surpassed £101/month. Since most of the medication given to these patients focused on the prevention of cardiovascular events, the authors argue that in patients with a limited life expectancy, the prescription of this medication is totally inappropriate and should be carefully reviewed [28].

Patient adherence to anti-diabetic medication remains a struggle, however, as some researchers imply that an important issue related to polypharmacy in diabetic patients is represented by the decreased compliance to anti-diabetic medication, which leads to an inadequate glycemic control, raising the risk of diabetes complications. The prescribing cascade is more frequent encountered in diabetic patients when a side effect of a drug is misinterpreted as a complication of the disease and, consequently, treated with another drug. The risk of medication error must not be neglected because it is associated with a higher risk of hospitalization and significant healthcare costs, especially in the elderly. Physicians should routinely monitor the diabetic patients for drug-drug, drug-food interactions, side effects, or even inappropriate medication. A multidisciplinary geriatric assessment is necessary in order to decrease the number of prescribed drugs. Pharmacists can be a useful chain link for detecting polypharmacy or drug interactions, as they can offer recommendations in order to simplify the therapeutic regimens and to detect the drug side effects. Different strategies have been suggested in order to control polypharmacy, such as using lists of inappropriate drugs or computer feedback after using Beers criteria. Small studies have demonstrated the benefits of these strategies in decreasing polypharmacy, but these must be confirmed in larger cohorts [29]. However, in high-income countries, it must be taken into account that polypharmacy has been part of the *armamentarium* that has led, in recent years, to a decrease in the percentage of diabetic patients who suffer from T2DM-related vascular complications. Moreover, due to a reduction in cardiovascular risk factors such as dyslipidemia or hypertension, cardiovascular mortality and all-cause mortality have both declined [30].

Balancing “the good” and “the bad” in the need for polypharmacy in patients with T2DM remains an unsolved struggle in daily medical practice and further studies are needed to explore this topic of paramount importance.

## 5. Conclusions

Polypharmacy should be an area of serious concern in type 2 diabetes mellitus, especially in the elderly. In our study, type 2 diabetes mellitus patients received more drugs than their non-diabetes mellitus counterparts and were exposed to more drug-drug and food-drug interactions. Strategies to reduce the prescription of unnecessary medication are needed and could improve the quality of life and adherence to therapy in patients with diabetes.

## Figures and Tables

**Table 1:** Differences in age, number of comorbidities and polypharmacy between patients with type 2 diabetes mellitus (T2DM) and non-diabetic subjects.

Variable	T2DM	Controls	*p*-Value
Age	69.19 ± 9.67	67.05 ± 14.4	0.0189
Comorbidities	10.35 ± 3.09	7.48 ± 3.59	0.0001
Prescribed drugs	7.81 ± 2.23	5.33 ± 2.63	0.0001
Drug-drug interactions	8.86 ± 5.76	4.98 ± 5.04	0.0003
Minor drug-drug interactions	1.22 ± 1.42	1.27 ± 1.89	0.8774
Moderate drug-drug interactions	7.08 ± 4.08	3.54 ± 3.77	0.0001
Major drug-drug interactions	0.56 ± 0.74	0.37 ± 0.77	0.1873
Food-drug interactions	2.63 ± 1.08	2.19 ± 1.42	0.0457

**Table 2:** Prescription patterns of anti-diabetic medication in our study group.

Drug or Drug Combination	Number of Diabetic Patients (%)
Metformin alone	13 (38.25)
Insulin alone	5 (14.71)
Metformin + Gliclazide	4 (11.76)
Metformin + Insulin	3 (8.82)
Metformin + Glimepiride	2 (5.88)
Gliclazide	2 (5.88)
Gliquidone	1 (2.94)
Metformin + Glibenclamide	1 (2.94)
Metformin + Pioglitazone	1 (2.94)
Gliclazide + Acarbose	1 (2.94)
Metformin + Gliclazide + Sitagliptin	1 (2.94)

**Table 3:** The most common drug-drug and food-drug interactions encountered in type 2 diabetes mellitus (T2DM) patients; *n* = number, % = percentage of the study group.

Type of Interaction	Drugs/Food	Controls (*n*)	Controls (%)
**Major Drug-Drug Interaction**	spironolactone-ramipril	4	6.35%
	spironolactone-candesartan	4	6.35%
	spironolactone-perindopril	3	4.76%
**Moderate Drug-Drug Interaction**	spironolactone-metformin	11	17.46%
	furosemide-metformin	10	15.87%
	aspirin-perindopril	9	14.29%
	furosemide-pantoprazole	8	12.70%
	metformin-perindopril	8	12.70%
	metoprolol-spironolactone	8	12.70%
	atorvastatin-pantoprazole	7	11.11%
	furosemide-digoxin	7	11.11%
	atorvastatin-clopidogrel	6	9.52%
	aspirin-amlodipine	6	9.52%
	metoprolol-amlodipine	6	9.52%
	furosemide-carvedilol	6	9.52%
	digoxin-metformin	6	9.52%
**Minor Drug-Drug Interaction**	aspirin-pantoprazole	7	11.11%
	digoxin-spironolactone	7	11.11%
	acenocoumarol-furosemide	7	11.11%
	acenocoumarol-atorvastatin	7	11.11%
	acenocoumarol-spironolactone	6	9.52%
**Food-Drug Interaction**	atorvastatin-grapefruit juice	24	38.10%
	perindopril-potassium salts	16	25.40%
	acenocoumarol-vitamin K rich foods	13	20.63%
	olmesartan-potassium salts	6	9.52%
	candesartan-potassium salts	6	9.52%
	ramipril-potassium salts	6	9.52%

**Table 4:** The most common drug-drug and food-drug interactions discovered in the control group (non-diabetic patterns); *n* = number, % = percentage of the control group.

Type of Interaction	Drugs/Food	Controls (*n*)	Controls (%)
**Major Drug-Drug Interaction**	spironolactone-candesartan	4	6.35%
	spironolactone-ramipril	3	4.76%
	spironolactone-perindopril	2	3.17%
	acenocoumarol-aspirin	2	3.17%
**Moderate Drug-Drug Interaction**	spironolactone-metformin	12	19.05%
	furosemide-metformin	10	15.87%
	aspirin-perindopril	8	12.70%
	metformin-perindopril	8	12.70%
	furosemide-pantoprazole	8	12.70%
	atorvastatin-pantoprazole	8	12.70%
	acenocoumarol-metformin	7	11.11%
	furosemide-metoprolol	7	11.11%
	furosemide-digoxin	7	11.11%
**Minor Drug-Drug Interaction**	aspirin-pantoprazole	9	14.29%
	acenocoumarol-furosemide	7	11.11%
	acenocoumarol-atorvastatin	7	11.11%
	metoprolol-aspirin	6	9.52%
	acenocoumarol-spironolactone	6	9.52%
	amlodipine-perindopril	5	7.94%
**Food-Drug Interaction**	atorvastatin-grapefruit juice	21	33.33%
	perindopril-potassium salts	15	23.81%
	acenocoumarol-vitamin K rich foods	9	14.29%
	olmesartan-potassium salts	5	7.94%
	candesartan-potassium salts	5	7.94%
	ramipril-potassium salts	5	7.94%
